# Community-Supported Shared Infrastructure in Support of Speech Accessibility

**DOI:** 10.1044/2024_JSLHR-24-00122

**Published:** 2024-09-26

**Authors:** Mark Hasegawa-Johnson, Xiuwen Zheng, Heejin Kim, Clarion Mendes, Meg Dickinson, Erik Hege, Chris Zwilling, Marie Moore Channell, Laura Mattie, Heather Hodges, Lorraine Ramig, Mary Bellard, Mike Shebanek, Leda Sarι, Kaustubh Kalgaonkar, David Frerichs, Jeffrey P. Bigham, Leah Findlater, Colin Lea, Sarah Herrlinger, Peter Korn, Shadi Abou-Zahra, Rus Heywood, Katrin Tomanek, Bob MacDonald

**Affiliations:** a University of Illinois Urbana-Champaign; b LSVT Global, Tucson, AZ; c Microsoft, Redmond, WA; d Meta, Menlo Park, CA; e Media Tuners LLC, Los Altos, CA; f Apple, Cupertino, CA; g Amazon, Seattle, WA; h Google, Mountain View, CA

## Abstract

**Purpose:**

The Speech Accessibility Project (SAP) intends to facilitate research and development in automatic speech recognition (ASR) and other machine learning tasks for people with speech disabilities. The purpose of this article is to introduce this project as a resource for researchers, including baseline analysis of the first released data package.

**Method:**

The project aims to facilitate ASR research by collecting, curating, and distributing transcribed U.S. English speech from people with speech and/or language disabilities. Participants record speech from their place of residence by connecting their personal computer, cell phone, and assistive devices, if needed, to the SAP web portal. All samples are manually transcribed, and 30 per participant are annotated using differential diagnostic pattern dimensions. For purposes of ASR experiments, the participants have been randomly assigned to a training set, a development set for controlled testing of a trained ASR, and a test set to evaluate ASR error rate.

**Results:**

The SAP 2023-10-05 Data Package contains the speech of 211 people with dysarthria as a correlate of Parkinson's disease, and the associated test set contains 42 additional speakers. A baseline ASR, with a word error rate of 3.4% for typical speakers, transcribes test speech with a word error rate of 36.3%. Fine-tuning reduces the word error rate to 23.7%.

**Conclusions:**

Preliminary findings suggest that a large corpus of dysarthric and dysphonic speech has the potential to significantly improve speech technology for people with disabilities. By providing these data to researchers, the SAP intends to significantly accelerate research into accessible speech technology.

**Supplemental Material:**

https://doi.org/10.23641/asha.27078079

Accessibility is a human right. The Universal Declaration of Human Rights specifies that “Everyone has the right of equal access to public service in his country” (United Nations General Assembly, 1949, Article 21), “everyone has the right to work” (Article 23), and “higher education shall be equally accessible to all on the basis of merit” (Article 26). Automatic speech recognition (ASR) currently provides millions of people with easy access to public services (over the telephone, on the internet, and via personal assistants), opportunities for improved work environments (e.g., novelist Richard Powers used ASR to dictate his National Book Award–winning novel, *The Echo Maker*; Powers, 2007), and education (e.g., by automatically transcribing recorded lectures and online classroom discussion). ASR enhances human rights, but it is not uniformly available to all: Many people with disabilities are unable to use ASR because their disability includes dysarthria.

In his history of the Americans with Disabilities Act, Lennard Davis notes that, like all human rights, accessibility is only received when those with the right to accessibility are willing to fight for their right (Davis, 2016). The history of speech technology is an extended study of the degree to which people with disabilities are willing to fight for the right of access. Fried-Oken proposed personalized ASR as an alternative computer interface technology for people with severe neuromotor disorders (Fried-Oken, 1985). Over the following decade, people with neuromotor disorders including cerebral palsy (CP) and traumatic brain injury repeatedly demonstrated the ability to achieve acceptably low error rates using personalized ASR, despite the presence of dysarthria as a co-occurrence of their motor disorders (e.g., Chang, 1993; Coleman & Meyers, 1991; Deller et al., 1991; Sy & Horowitz, 1993).

Research on ASR for dysarthric speech was facilitated in the first decades of the 21st century by the widespread distribution of three small corpora of dysarthric speech designed for training and testing ASR. The Nemours database of dysarthric speech (Menendez-Pidal et al., 1996) contains 74 sentences read by each of 10 male speakers with dysarthria. The Universal Access speech corpus (UA-Speech; Kim et al., 2008) contains 765 isolated words (455 distinct) read by each of 19 speakers with dysarthria as a symptom of CP. The TORGO database of acoustic and articulatory speech (Rudzicz et al., 2012) contains read sentences, isolated words, and assessments of speech intelligibility produced by seven speakers with dysarthria associated with CP or amyotrophic lateral sclerosis (ALS). The importance of shareable data can be emphasized by studying the history of word error rates on the UA-Speech corpus (see Figure 1). The first reported word error rate using the standard test portion of UA-Speech was 69.2% (Sharma, 2008). Over the succeeding 4 years, the same team reported successively improved results (i.e., reduced error rates): 66.7% in 2010 (Sharma & Hasegawa-Johnson, 2010) and 58.7% in 2012 (Sharma, 2012). In 2014 and 2015, new improved results were reported by a different team who reported word error rates of 40.5% in 2014 (Christensen et al., 2014) and 34.9% in 2015 (Sehgal & Cunningham, 2015). From 2018 until 2023, new improved results were reported on the UA-Speech test set every year, by teams from universities in China, Germany, and the United Kingdom: 32.2% (Yu et al., 2018), 27.9% (Xiong et al., 2019), 26.6% (Liu et al., 2020), 25.2% (Liu et al., 2021), 22.5% (Baskar et al., 2022), and 17.8% (Geng et al., 2023).

**Figure 1.**
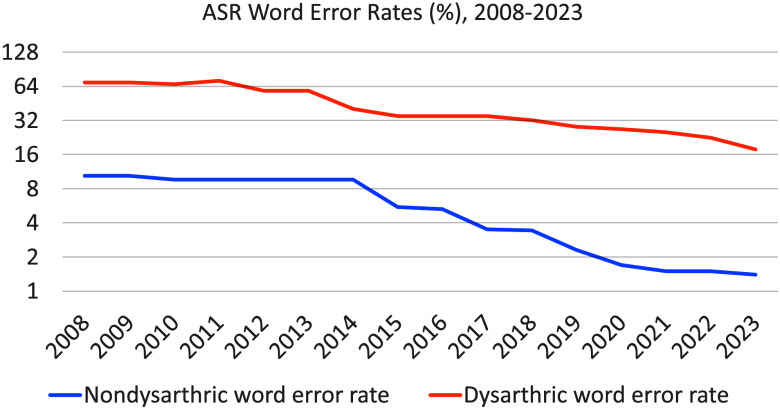
Best published word error rates (%), up to each specified year, of automatic speech recognizers tested on standard, widely available corpora of dysarthric speech (orange, top curve) and nondysarthric speech (blue, bottom curve). ASR = automatic speech recognition.

UA-Speech, Nemours, and TORGO made possible continuous and impactful advances in dysarthric speech recognition that would have been improbable without these corpora, but it must be noted that, while the lowest dysarthric ASR error rates dropped by a factor of 3 between 2008 and 2023, the lowest error rates for people without dysarthria dropped by a factor of 5 over the same period (see Figure 1 for a summary of results reported using the standard Switchboard and LibriSpeech Test-Clean corpora; Amodei et al., 2016; Dahl et al., 2013; Han et al., 2017; Hwang et al., 2023; Lüscher et al., 2019; Nguyen et al., 2005; Panayotov et al., 2015; Park et al., 2020; Schlüter et al., 2010; Xu et al., 2021; Yin et al., 2018). Part of the reason for the rapid improvements in nondysarthric ASR during this period of time is the rapid improvement in the availability of data designed for standardized, repeatable experiments in the training and testing of nondysarthric ASR. In 2008, the largest widely available English-language data set with clearly defined benchmark training and test sets was Switchboard (Godfrey et al., 1992), which contains 300 hr of speech produced by 500 speakers. In 2015, new standard training and test sets were provided in the LibriSpeech corpus (Panayotov et al., 2015), which contains 960 hr of speech produced by 1,100 speakers. ASR error rates on LibriSpeech are significantly lower than on Switchboard in part because it is much bigger and in part because the audio is far less noisy. Project Euphonia demonstrated that similar large advances are possible for disordered speech, if the training and test data set is large, diverse, and well curated (Macdonald et al., 2021; Tobin & Tomanek, 2023). However, Project Euphonia data cannot be distributed as the original consent does not include this scenario. The success of Project Euphonia suggests that an important part of the reason that dysarthric ASR improved less rapidly than nondysarthric ASR, from 2008 to 2023, is that there were no improvements in the widespread availability of data for standardized, repeatable training and testing of dysarthric speech: The largest English corpus designed according to this criterion was the UA-Speech corpus (22 hr of speech by 16 speakers) from 2008 to 2023. Given the limitations of the existing corpora, we designed the Speech Accessibility Project (SAP), which aims to collect, curate, and distribute a data set of atypical speech that contains a large-enough number of speakers, and a large-enough quantity of transcribed speech audio, to permit the development of effective ASR for people with disabilities.

## Background

Results in the literature suggest that, to effectively encourage the training and testing of ASR for people with speech disabilities, four criteria are desirable:

1. The training corpus should contain a large amount of speech from many participants.
2. Participant voices in the training corpus should be similar to the voices of participants who will use the developed ASR.
3. Participant privacy must be respected while enabling both researcher and commercial use cases.
4. Each entry should be distributed with annotations indicating dysarthria severity.

The first criterion required of an ASR corpus is that it should contain a large amount of speech from many participants. For example, LibriSpeech (960 hr of speech) has resulted in much lower ASR error rates than are possible using Switchboard (300 hr; Godfrey et al., 1992; Panayotov et al., 2015). Acquiring a large amount of speech requires designing a set of prompts that is large enough and that is varied and interesting enough to maintain participant interest during the task. The number of required prompts can be estimated based on durations of sentences in published corpora, which vary from 3 s in the Texas-Instruments Massachusetts Institute of Technology phonetic speech corpus (Garofolo et al., 1993; Lopes & Perdigao, 2011) to 5 s in Switchboard (Godfrey et al., 1992; Hamaker et al., 1998); at 3 s per sentence, each hour of speech is equivalent to 1,200 sentences. Additionally, the number of participants recruited for the project should be as large and diverse as possible because ASR is best able to transcribe the speech of a test speaker if its training corpus contains similar speech: In one study, for example, English speakers with no disability had word error rates varying from 1.1% to 7.4%, depending on the similarity of their individual speech patterns to the patterns learned by the ASR from its training corpus (Gauvain et al., 1994).

Second, the corpus should contain a range of variability that represents, as accurately as possible, the range of variability on which the ASR will be tested. The target of the SAP is the use of speech to provide “access for people with physical, sensory, or cognitive disabilities” to “computers, telecommunications equipment, … software, websites, information kiosks and … electronic documents” (U.S. Access Board, 1986). Speech-language pathologists have developed standard terminology for disabilities of speech, voice, and language (e.g., dysarthria, dysphonia, aphasia, and speech sound disorder; flaccid, spastic, ataxic, hypokinetic, and hyperkinetic dysarthria; Darley et al., 1969), but people with speech, voice, and language disorders are rarely familiar with these labels. Therefore, for purposes of recruitment, the SAP is focused on etiologies that are well understood by people without specialist training in speech and language pathology. The 2023-10-05 Data Package contains the speech of people with Parkinson's disease (PD), including idiopathic PD and three of the most common atypical parkinsonism categories described by Levin et al. (2016). Hypokinetic dysarthria is the most common dysarthria type symptomatic of PD (Kim et al., 2011; Levy et al., 2020; Ramig et al., 2018), although atypical parkinsonism may have other symptoms: Progressive supranuclear palsy may present with a combination of hypokinetic, spastic, and ataxic dysarthria (Kluin et al., 2001), while ataxic dysarthria is the most common dysarthria type symptomatic of multiple systems atrophy (Kim et al., 2011).

Third, for a corpus to successfully encourage the development of effective ASR, its intellectual property status and distribution policies should encourage its use for projects ranging from undergraduate thesis projects to large-scale corporate ASR development. There is a subtle but important conflict between the goal of free distribution and the goal of maximum participation: Many participants will not contribute their speech to a database that can be replicated and reused by anybody for any reason because they disapprove of some particular uses that might be made of such a corpus or are concerned about biometric reidentification and other potential privacy or safety harms. Prior to the SAP, the conflict between participant privacy and broad distribution was often resolved (e.g., by the UA-Speech [Kim et al., 2008] and TalkBank [MacWhinney, 2007] corpora) using a license that forbids commercial uses, based on the belief that most uses that violate participant privacy are commercial uses. A noncommercial license is, however, incompatible with the goal of developing commercial products that will facilitate speech accessibility. 

Fourth, studies using the UA-Speech corpus have demonstrated that annotations of dysarthria severity can be used to reduce the error rate of dysarthric ASR (Geng et al., 2023). Different corpora have annotated dysarthria severity in different ways, including categorical ratings (mild, moderate, severe), gradient (intelligibility), and multidimensional (differential diagnostic pattern dimensions).

## Method

The SAP was approved by the University of Illinois institutional review board (protocol #23183). All participants indicate informed consent using an online form. Project recruitment materials encourage people to volunteer for participation if they consider themselves to have any form of PD, regardless of whether or not they believe themselves to have consequent speech impairment. Each potential participant provides their contact information on the project webpage and specifies that they consider themselves to have PD. The potential participant is then contacted by a project mentor, who is a speech-language pathologist with expertise in PD. The mentor decides whether or not to include the participant in the study on the basis of the mentor's assessment of speech disability: After a brief individualized speech screening, if the mentor rates the participant's speech intelligibility or voice naturalness impairment at any level higher than Level 1 (out of 7) on an etiology-specific impairment scale on which label ordinate 1 refers to speech or voice within normal limits and label ordinate 7 refers to the most severe speech or voice impairment (e.g., for PD, the dysarthria differential diagnostic scales of Darley et al., 1969, are used), then the participant is invited to participate in the study. Since the SAP is focused exclusively on ASR rather than medical diagnosis, the project attempts no independent confirmation of the patient's self-reported PD diagnosis. The mentor then ensures that the participant understands the consent form before approving their participation in the project. The consent form specifies that data will be distributed for both commercial and noncommercial use but that (a) no researcher using these data will seek to identify any individual participant; (b) participant demographic information including race, ethnicity, age and gender will be collected by the project but will be released only in the form of aggregate statistics; (c) researchers using the data will employ safeguards to prevent data theft; and (d) if a participant later chooses to remove their own data from the data set used by researchers, then all researchers will be told the identification code of the removed participant, and agree to remove that participant's data from any future experiments.

Mentors screen the participants on quality of internet connection and ability to reduce background noise when recording. Motor and cognitive functions are screened to ensure participant can manage the data collection. If needed, a helper (spouse, roommate, and adult child) can be trained to assist.

Mentors train participants on the software. Mentors encourage participants to record on a microphone and from a consistent mic-to-mouth distance that is appropriate to a speakerphone call or a video conference, and to use a naturalness of voice and speech that is appropriate to speaking to a family member. For this purpose, participants are told that “We know that you can make yourself more intelligible by pretending that you're performing for an audience, and there is a tendency for people to do that when they are speaking to a computer, but that's exactly what we don't want for this study. We want to train the computer to understand your at-home voice, that you would use if you had just come home from a long day and you were relaxing on the couch with your spouse or your family.” Participants are then asked to record several prompts to ensure naturalness, and ease with the platform, and to ensure that the recording software succeeds. When the participant feels confident with the task, the mentor and participant end their initial meeting. After the initial meeting, the participant records speech on a schedule of their own choosing, using their own equipment in their own home or comfortable environment. Participants are encouraged to contact their mentor if the participant needs assistance later in the project; mentors also take the initiative to perform brief quality assurance checks on the audio fidelity and speech severity of samples and to follow up periodically to help maintain participant engagement with the project. As of October 5, 2023, 746 people with PD had registered for meetings with project mentors. Of these, 283 had been approved for participation, and 253 had recorded data; the remaining 463 were judged to have speech and voice characteristics within normal limits (Level 1 on the perceptual scales of Darley et al., 1969) and were therefore not accepted for participation in the SAP.

Each participant in the SAP is asked to read or repeat 350–400 phrases and to respond to 50–80 prompts for spontaneous speech with responses of approximately four sentences each. The majority of participants complete the protocol; the 2023-10-05 Data Package includes 74,467 recorded responses, an average of 406 per participant. Read or repeated speech includes 300–350 digital assistant commands per participant (drawn from a superset of 2,130 digital assistant commands) and up to 100 sentences from novels (drawn from a set of 27,800 novel sentences, simplified from texts used in Multilingual LibriSpeech). These prompts are read from the screen if the participant is able to read and has sufficient visual acuity to read prompts on screen; if not, the participant hears each prompt read by a synthetic voice and repeats it after hearing it (all of the 211 participants in the 2023-10-05 Data Package and all of the 42 participants in the associated test set were able to read from the screen; none relied on the synthetic voice). At the time of this writing, there are three distinct sets of prompts: (a) a set of prompts designed for a standardized 10th-grade literacy level, including prompts for spontaneous speech; (b) a set of prompts designed for a standardized sixth-grade literacy level, including prompts for spontaneous speech; and (c) a set of prompts in which prompts for spontaneous speech are replaced by read isolated words, designed for people who express discomfort with spontaneous speech because of the severity of their speech disability (all of the 253 participants in the 2023-10-05 Data Package and associated test set read the prompts designed for a 10th-grade reading level; none used the other two sets of prompts). Table 1 shows examples of digital assistant commands, novel sentences, and spontaneous speech prompts designed for participants with 10th- and sixth-grade reading levels.

**Table 1.** Examples of prompts designed for participants with Parkinson's disease (longer sentences, with more complex vocabulary, involving 20th-century concepts) and for participants with Down syndrome (shorter sentences, less complex vocabulary, focused on 21st-century concepts).

Prompt type	Longer prompt sentences, e.g., for participants with Parkinson's disease	Shorter prompt sentences, e.g., for participants with Down syndrome
Digital assistant commands	Add meat for grilling, precut vegetables, teriyaki sauce, and bamboo skewers to the shopping list.	Add meat and vegetables for grilling to shopping list. Remember that it's time to change the air filter.
Sentences from novels	“Oh, I'm not going to call you Stubby Toes anymore!” laughed Sister.	None
Spontaneous speech prompts	Please explain the steps to change a tire on a car. Please explain the steps to mail a letter.	Explain how to search something on the internet. Explain the steps to send a text message.

*Note.* Prompt types include digital assistant commands (read), sentences from novels (read), and spontaneous speech prompts (to which participants are expected to respond with spontaneous speech).

All utterances in the SAP are audited by human transcribers with experience listening to dysarthric speech (e.g., clinicians and students of speech-language pathology). Spontaneous speech is transcribed verbatim. Each participant's speech was initially transcribed by a student annotator. The transcription was then finalized after discussion between the annotator and the third author. Transcriptions of read and repeated speech are initialized using the prompt text, then edited by a human transcriber so that the transcription matches what the participant actually said.

One or more speech-language pathologists trained in the use of the differential diagnostic patterns of speech and voice used them to rate each of 30 read sentences per participant. Affirmation of the classic speech symptoms for PD was made by intersite agreement among professional speech clinicians in Illinois and LSVT Global. Rated samples include 15 from the first block of prompts and 15 from the last block of prompts, to permit the detection of any change during the period of participation. Ratings use the 38 dimensions of speech and voice proposed in Darley et al. (1969), with a small number of modifications. First, as recommended by Yorkston (2010), “Bizarreness (overall)” is replaced by “Naturalness (overall),” with the understanding that higher scores represent speech that differs from the prosodic and rhythm patterns expected by the listener. Second, four additional dimensions are added: “Impaired loudness control,” “Impaired emphasis,” “Impaired pitch control,” and “Slow rate,” based on the recommendations in Darley et al. (1975). Finally, a dimension called “Other” is included, for the annotation of unusual perceptual features that are considered salient by the annotator and that are not well captured by any existing dimension (e.g., diplophonia).

Although early accessibility advocates demonstrated accurate ASR for personalized models, SAP aspires to enable speaker-independent, out-of-the-box ASR to be useful for these populations; therefore, participants in the training set, test set, and development (dev) set are nonoverlapping. The machine learning community commonly depends on availability of both a dev set that can be used to assess performance multiple times during training and a test set that is only used one time as a final model assessment. The 2023-10-05 Data Package is divided into ASR training and dev subsets on the basis of the text prompts read by participants, and an associated test set is not distributed with the Data Package because it is reserved for future experiments. Each participant was assigned prompts from one of 10 standard prompt lists. Participants who read List 5 are assigned to the dev set (10% of participants), participants who read Lists 9–10 are assigned to the test set (20% of participants), and all others are assigned to the training set (70% of participants). In this way, it is possible for the dev and test set to each contain some texts that never appear in the training set; the cost of maintaining this separation is that the dev and test sets consume a somewhat larger fraction of the corpus than is typical of recent ASR corpora (e.g., Panayotov et al., 2015). The 2023-10-05 Data Package includes the speech of 211 participants with PD: 190 speakers in the training set (151.47 hr of speech) and 21 in the development set. Additional 42 speakers contributed to the test set, which is not distributed with the data package. The utterances from each participant in the dev and test sets are further subdivided into shared and unshared subsets, depending on the text of the utterance. Unshared utterances (42% of utterances by design, although the percentage may vary due to variability in participant completion rates) are of two types: digital assistant commands that are unique to the participant's prompt list (31% of utterances) and spontaneous speech (11% of utterances, although these utterances tend to be longer than read speech). Shared utterances (58% of utterances) are of four types: digital assistant commands or sentences from novels that are read twice by all participants (18% of utterances), digital assistant commands that are each read once by all participants (6%), digital assistant commands that are each read by 20% of participants (16%), and sentences from novels that are each read by 0.3% of participants, chosen at random (18%).

As an initial baseline for future experiments in dysarthric speech recognition, three ASR systems were tested. Details of the three systems are shown in Table 2: LibriSpeech-100 h, LibriSpeech-960 h, and a system fine-tuned with the training set from the SAP 2023-10-05 Data Package. The LibriSpeech-100 h system is an open-source implementation of the wav2vec 2.0 base model (Baevski et al., 2020), pretrained by its authors using unlabeled speech, then fine-tuned using 100 hr of labeled LibriSpeech data. The LibriSpeech-960 h system is similar but was fine-tuned using 960 hr of data, rather than 100 hr. Trained parameters for both of these systems were downloaded from the fairseq open-source repository and not further trained. The only system fine-tuned using data from the SAP is the third system listed in Table 2; this was the same wav2vec 2.0 base model used in the other two systems, but instead of being fine-tuned on LibriSpeech, all of its parameters were fine-tuned using the training set of the SAP 2023-10-05 Data Package on hardware provided by the National Science Foundation (Kindratenko et al., 2020). Training continued until performance peaked on the dev set and was then tested using the shared and unshared subsets of the test set. Speakers in the training, dev, and test sets do not overlap (i.e., no test speakers were observed by the system during training). Test speakers read some prompts that were also read by training speakers, and these sentences were collected in the shared subset. Test speakers also read some prompts that were not read by any training or development speakers; these sentences were collected in the unshared subset. Preprocessing steps include audio resampling, text preprocessing using Nemo text normalization (Zhang et al., 2021), and punctuation removal.

**Table 2.** Word error rates (%) of two open-source models (LibriSpeech-100 h and LibriSpeech-960 h) and of a system fine-tuned using the SAP training set.

Foundation model	Fine-tuned on what data?		Word error rates (%)		
			All test data	Shared	Unshared
wav2vec 2.0 base	LibriSpeech training set: audiobook narrators	100 h	45.15	52.29	42.53
		960 h	38.76	45.39	36.33
	Speech Accessibility training set: speakers with PD	151 h	**18.72** **	**5.18** **	**23.69** **

*Note.* Test speakers and training speakers do not overlap. Shared test set consists of prompt texts that were also read by training speakers. Unshared test set consists of prompt texts not read by training speakers. Lowest word error rate in each column is in boldface. SAP = Speech Accessibility Project; PD = Parkinson's disease.

** Denotes word error rates that are significantly lower than those of the LibriSpeech-960 h model, at a significance level of *p* < .001 (MAPSSWE test computed using NIST sc_stats: Pallet et al., 1990).

## Results

The 2023-10-05 Data Package contains the speech of 211 people with PD (the train and dev subsets described in this article). This number is approximately an order of magnitude larger than existing corpora designed for the development of dysarthric ASR.

After completing all recordings, participants were asked, “Do you consider U.S. English to be your native language?” Although nonnative speakers were not excluded from participation, all participants included in the 2023-10-05 Data Package, and all participants in the associated test set, answered “Yes” to this question.

### Differential Diagnostic Patterns

**Figure 2.**
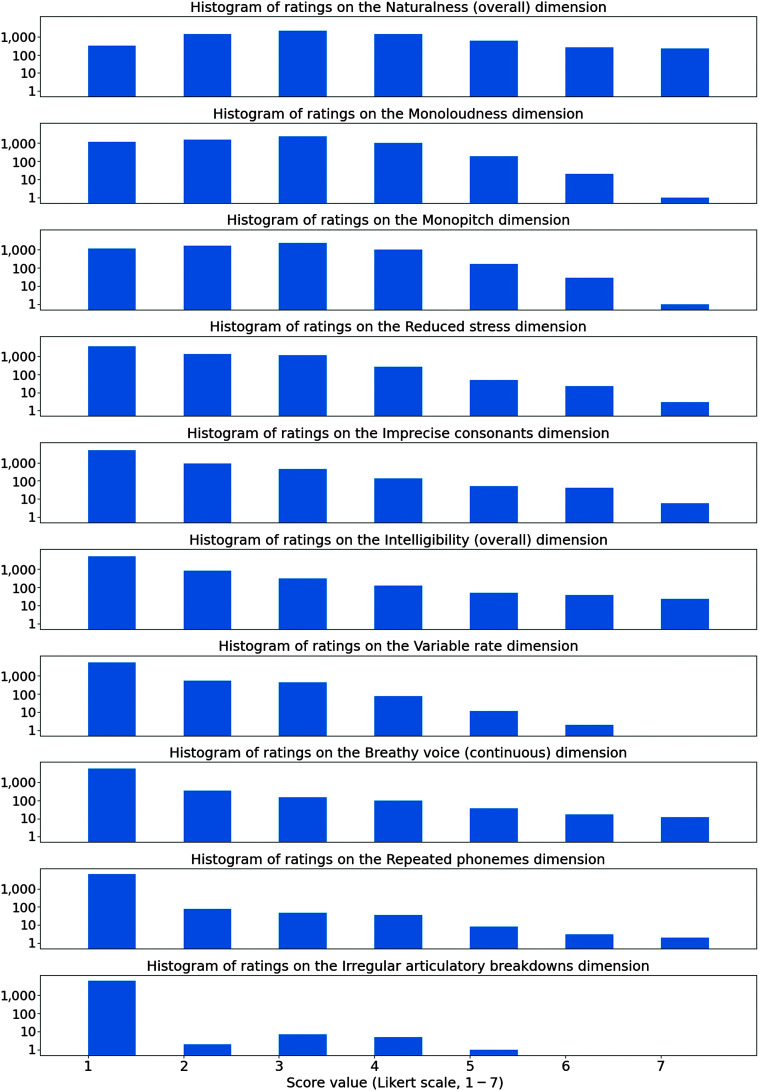
The differential diagnostic pattern dimensions in the study of Darley et al. (1969) are a set of 41 Likert scales, each describing a dimension of disability audible in an utterance. A total of 5,342 utterances in the 2023-10-05 SAP data package (an average of 25.3 per participant) were rated using differential diagnostic pattern dimensions. This figure shows histograms of those 5,342 utterances, as rated using 10 of the differential diagnostic pattern dimensions.

We present rating histograms for several of the differential diagnostic pattern dimensions to provide examples representing the types of speech produced by the 191 participants in the train subset (see Figure 2). The top 7 histograms in Figure 2 show the seven differential diagnostic scales with the highest averages in this corpus. Each of these histograms shows the distribution of ratings received by rated speech recordings; a total of 5,342 recordings in the 2023-10-05 Data Package are rated (an average of 25.3 per participant). On all of these scales, a rating of 1 means *within normal limits*, a rating of 2 is *mild impairment*, ratings of 3–4 represent *moderate impairment*, and ratings of 5–7 represent *severe impairment*. The scale with the highest average rating was *Naturalness*. Only 320 recordings received a Naturalness rating of 1, and no single participant received a Naturalness score of 1 on all of their productions, suggesting that all participants had some type of speech impairment.

The next highest average scores were on the scales *Monoloudness* and *Monopitch*. Participants with ratings above 1 on the scales *Monoloudness* or *Monopitch* would be categorized, under the rating scheme of (Darley et al., 1969), as exhibiting hypokinetic dysarthria. On these two scales, 18% of waveforms had a rating of 1 (*within normal limits*), 25% had a rating of 2 (*mild dysarthria*), 53% had ratings of 3 or 4 (*moderate dysarthria*), and only 3% had ratings of 5–7 (*severe dysarthria*).

As dysarthria is often associated with a reduction in speech intelligibility, the perceptual scale *Intelligibility* might be considered the most direct measure of dysarthria severity. Ratings on this scale tended to be lower than those on the *Monoloudness* and *Monopitch* scales. Ratings on this scale correlated with ratings on the scale *Imprecise consonants*. On both *Intelligibility* and *Imprecise consonants*, 76% of recordings received ratings of 1 (*within normal limits*), 14% received ratings of 2 (*mild dysarthria*), 8% received ratings of 3–4 (*moderate dysarthria*), and only 1.5% received ratings of 5–7 (*severe dysarthria*).

The last three scales shown in Figure 2 demonstrate unusual speech patterns that each occur in a minority of the recorded samples. Utterances with high scores for “Breathy voice (continuous)” are often whispered, or nearly whispered, which may have a physiological correlate to bowed vocal folds, a common presentation in PD (Smith et al., 1995). Utterances with high scores for “Repeated phonemes” exhibit stuttering-like disfluencies, sometimes quite severe. Utterances with high scores for “Irregular articulatory breakdowns” exhibit characteristics of ataxia.

### Sample Spectrograms and Response Texts

**Figure 3.**
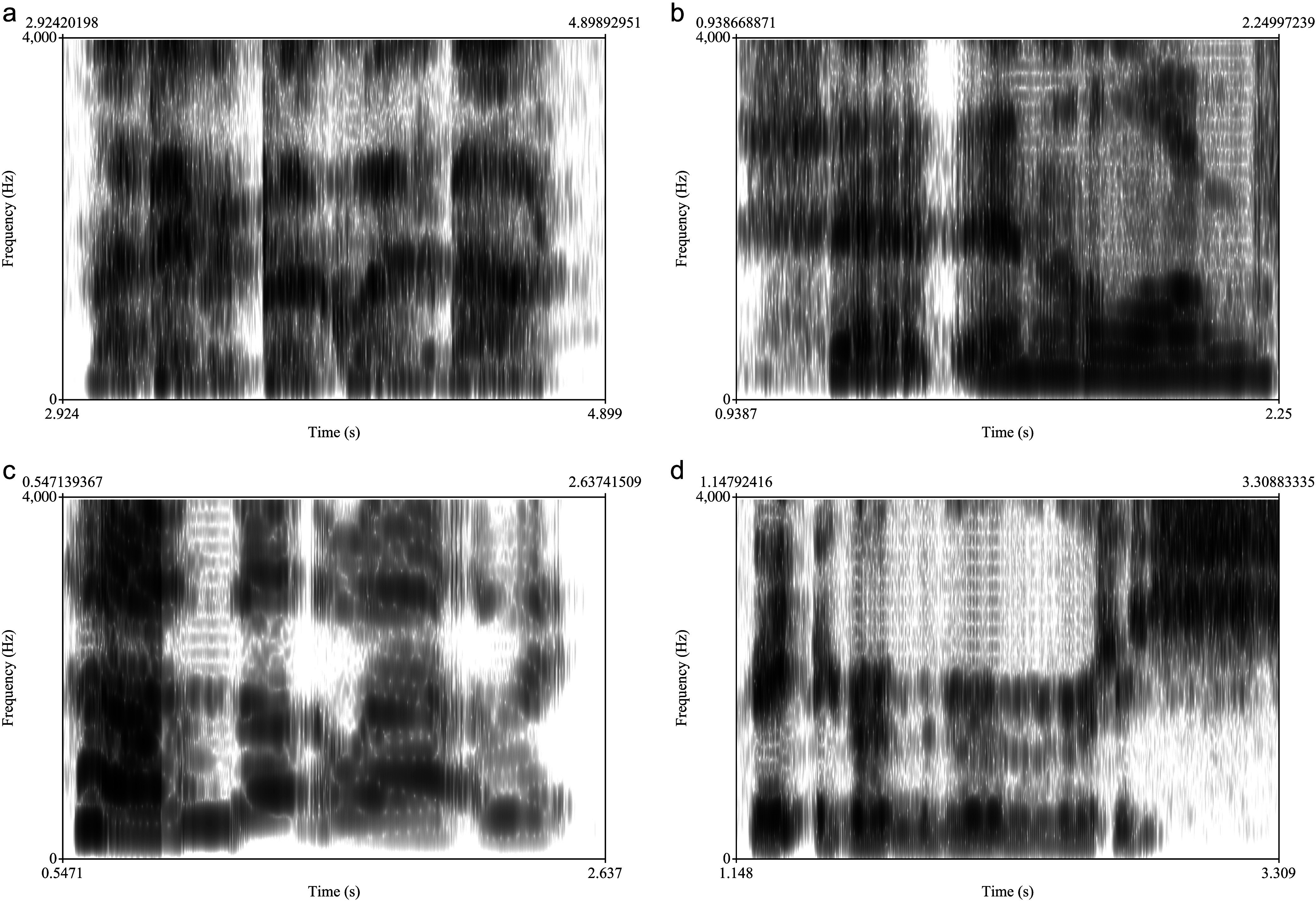
Examples of utterances with low, moderate, and high intelligibility loss. (a) Utterance with low intelligibility loss, but highly breathy voice: “My favorite book … ” (b) Utterance with moderate intelligibility loss, glottalized on the first word: “Set an alarm … ” (c) Utterance with high intelligibility loss: “How's the traffic … ” Note the drop in loudness by 20 dB following the first syllable, the sonorance of the two fricatives, and the reduction of the /r/ in “traffic” to a /w/. (d) Utterance with high intelligibility loss and high prevalence of repeated phonemes: “Create a (g-) grocery sh-.” Note approximately 10 attempts to produce the /g/ in grocery, from 1.56 s to 2.50 s.

Figure 3 shows spectrograms of participants whose average “Intelligibility (overall)” scores were (a) low, (b) moderate, and (c) high (indicating low, moderate, or high degree of intelligibility impairment, respectively). Figure 3a is a spectrogram with low intelligibility impairment, but whose naturalness impairment is somewhat higher, because the speech is whispered. Figure 3b is a spectrogram with moderate intelligibility impairment, but with higher naturalness impairment because of creaky voice. Figure 3c is a spectrogram with high intelligibility impairment associated with consonant misarticulation and high loudness variation. Figure 3d is a spectrogram with high intelligibility impairment associated with an unusually high level of phoneme repetition.

**Figure 4.**
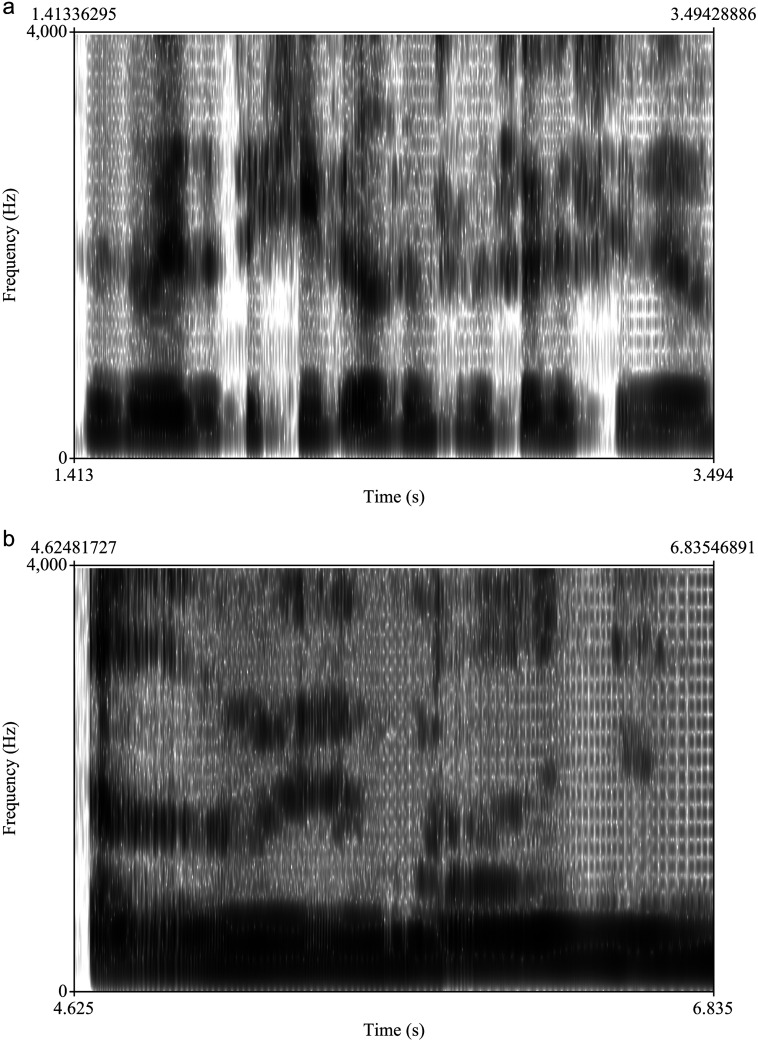
Examples of unusually fast and unusually slow speech. (a) Unusually fast, hypoarticulated speech: “That is the way that they keep elephants at the circus, you know” (16 canonical syllables, 14 produced syllables, 2 s). (b) Unusually slow, creaky speech: “Set a reminder … ” (four syllables in 2 s).

Speaking rate also varies considerably within the corpus. The spectrogram in Figure 4a shows 16 canonical syllables in 2 s; the spectrogram in Figure 4b shows four syllables in 2 s.

Participants demonstrated considerable variability in their spontaneous responses to prompt questions. For example, consider the responses by two different participants to the prompt “Please explain the steps to making breakfast for 4 people,” provided in Supplemental Material S1. Participant 4B8AAE89-754D-4C6E-F7DA-08DB65F35C8B describes an elaborate breakfast of Eggs Florentine, involving the creation of hollandaise sauce the day before. By contrast, participant C904EF6B-7796-4D27-DB5E-08DB263BD57D says that “The best way to make breakfast for four people is to ask them what they want, and then order it from a take out restaurant.” Although their responses differ considerably in both length and vocabulary, response texts suggest that both participants were fully engaged in the process of contributing speech samples to the corpus.

Responses also differ considerably in the use of proper nouns. Proper nouns are known to be challenging for ASR (e.g., Laurent et al., 2014; Peyser et al., 2020), so, in order to encourage the development of accessible devices that are able to correctly recognize proper nouns, read prompts were designed to include many proper nouns and prompts for spontaneous speech were designed to encourage participants to respond with proper nouns. For example, in response to the prompt “Tell me about your favorite musician,” one participant responded: “One of my favorite bands. (uh) I would say (um) Keith Jarrett Trio. With (uh), oh boy. Keith Jarrett, Jack DeJohnette, was it Charlie Haden?” Another responded: “Blake Schwarzenbach from Jawbreaker,” while a third responded: “I enjoy listening to Francine Reed, Marni Nixon.” In all three cases, transcribers verified correct spelling of the names using online resources.

### Preliminary Results Using ASR

Results of ASR experiments are shown in Table 2. First results indicate

(1)  The increase of typical speaker training data in LibriSpeech-960 h compared to LibriSpeech-100 h decreases the error rate on the test set associated with the SAP 2023-10-05 Data Package from 45.2% to 38.8%, without further fine tuning.
(2)  A wav2vec 2.0 base system fine-tuned using the SAP 2023-10-05 Data Package training set is able to recognize speech in the shared test set associated with the SAP 2023-10-05 Data Package with an error rate of only 5.2%. This error rate is too optimistic, however, because the shared subset consists of prompt texts that also occur in the training set. The ASR has apparently learned to expect the prompt texts in the training corpus and therefore recognizes the same texts in the shared set with a very low error rate.
(3)  A wav2vec 2.0 base system fine-tuned using speakers with PD (the 2023-10-05 training set) can recognize a different set of texts, spoken by a different set of speakers with PD (the unshared test set associated with the 2023-10-05 Data Package) with an error rate of 23.69%. This error rate is less than two thirds the error rate achieved on the same data by a system fine-tuned using 960 hr of speech from people without PD.

**Figure 5.**
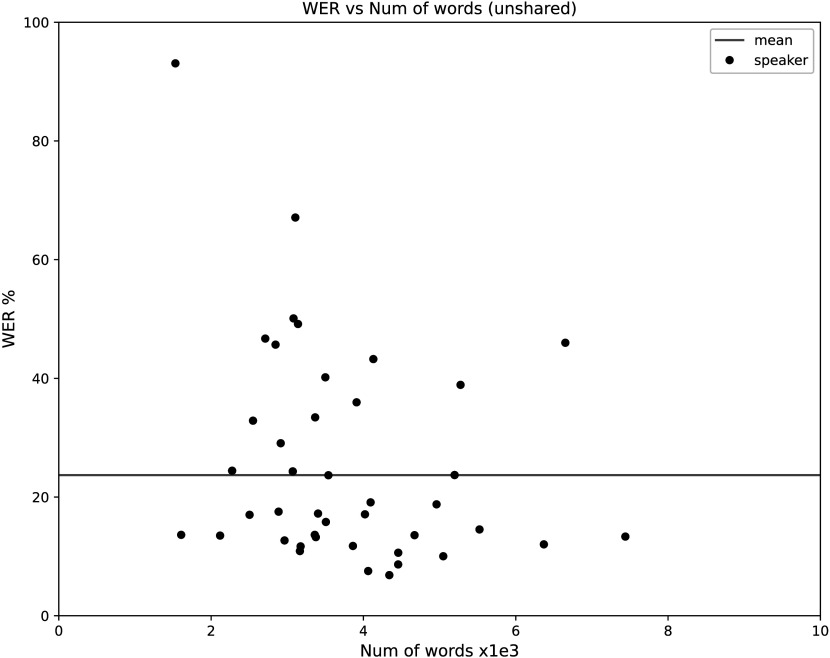
Average word error rate on the unshared prompts spoken by each person in the test corpus, plotted as a function of the number of words in the corresponding reference transcript. WER = word error rate.

Figure 5 is a scatter plot of the average ASR error rate for the unshared speech samples of each speaker in the test corpus (ordinate), plotted as a function of the total word count in the reference transcripts of that person's unshared speech (abscissa). As shown in Figure 5, different speakers have very different error rates, and the differences are not significantly predicted by the amount of test data available. Of the 42 speakers in the test set, 25 have error rates below the corpus average of 23.69%, 15 have error rates between 23.69% and 50.10%, and two speakers have error rates that are much higher (67.10% and 93.08%, respectively).

## Discussion

The SAP has been designed as a shareable data set that will significantly accelerate research in speech accessibility. Aspects of the data set that have the potential to greatly accelerate accessibility research include its variety of lexical and stylistic content and its variety of speaker characteristics, including a variety of types of speech impairment. Although the 2023-10-05 Data Package contains only the speech of people with PD, closer examination of the corpus demonstrates that there is already substantial variation in the types of speech disability represented. Some aspects of that variability are surfaced by a closer examination of the results of preliminary ASR experiments.

Table 3 shows how the error rate varies depending on the type of speech prompt: digital assistant commands (read speech), sentences from novels (read speech), or prompts for spontaneous speech. A system fine-tuned using LibriSpeech achieves considerably lower error rate when recognizing sentences from novels than when recognizing any other type of speech, possibly because these sentences in the SAP prompt set are simplified and regularized versions of sentences that also occur in LibriSpeech. The system fine-tuned using SAP data achieves roughly 5% error rate on either of the prompt types that occur in shared test data and roughly 24% error rate on any of the three prompt types that occur in unshared test data. When averaged across the entire test corpus, the error rate of this system is lowest on digital assistant commands, presumably because the shared texts make up a larger fraction of the digital assistant commands than of any other prompt type.

**Table 3.** Word error rate (%) of two speech recognizers on three types of prompts.

Type of prompt	Number of utterances in SAP test set	Duration (hours) of SAP test set	Word error rate (%) Fine-tune: L960, Test: SAP test set	Word error rate (%) Fine-tune: SAP training set Test: SAP test set		
				Shared	All	Unshared
Digital assistant commands	11,966	17.16	50.53	5.28	**12.62**	25.50
Spontaneous speech prompts	1,948	13.68	37.04	—	23.05	**23.05**
Sentences from novels	4,002	9.00	**25.43**	**4.81**	19.60	23.95

*Note.* Recognizers: one fine-tuned using LibriSpeech-960 h (L960), one fine-tuned using the Speech Accessibility Project (SAP). Types of prompts: digital assistant commands (read), spontaneous speech prompts, and sentences from novels (read). Shared prompts are read by both test and training speakers. Unshared prompts are read by only test speakers. All = Shared + Unshared. Lowest error rate in each column is in boldface.

## Future Directions

Recruitment of participants for the SAP is ongoing. Participants with Down syndrome (DS), CP, ALS, and speech disabilities related to cerebrovascular accident (CVA) have been specific targets of recruitment since November 2023. The goal of current recruitment is to enroll 400 participants from each of the five etiologies, for a total of 2,000 participants. These etiologies are targets of ongoing recruitment because they are correlated with symptoms that are quite diverse and different from the symptoms of PD:

- CP: For CP, the overall muscle tone diagnosis (spastic, athetoid, or mixed) often correlates with the perceptual attributes of dysarthria (spastic, ataxic, or mixed; Ansel & Kent, 1992).
- ALS: The dysarthria symptoms of ALS have been described as “truly a mixed dysarthria” (Darley et al., 1969), with both flaccid and spastic perceptual characteristics.
- DS: Difficulties with speech intelligibility in DS emerge from a combination of motor delays; craniofacial and laryngeal dysmorphologies, including morphology of the tongue and jaw; and phonological or articulatory disorders that are characterized by “inconsistent errors and possibly increased variability at the acoustic level” (Kent & Vorperian, 2013).
- CVA: Speech disability following CVA varies, with 70% of patients showing some dysarthria at the time of hospital discharge (Vidović et al., 2011), of which the plurality exhibit symptoms of unilateral upper motor neuron dysarthria (Kim et al., 2011).

Focus group data from these four new etiologies provided insight into the differential diagnostic dimensions that are currently being included in annotating their speech patterns. The original differential diagnostic patterns established by Darley et al. (1969) included bulbar palsy (e.g., flaccid dysarthria), pseudobulbar palsy (e.g., spastic dysarthria), ALS, cerebellar disorders (e.g., ataxic dysarthria), parkinsonism, dystonic hyperkinetic dysarthria, and choreic hyperkinetic dysarthria. Multiple sclerosis was added later (Darley et al., 1975). For SAP to achieve the goal of establishing severity annotations to facilitate ASR while maintaining continuity in the annotation process, considerations were made for the DS and CVA participants. The speech patterns of individuals with DS continue to be investigated with continued questions about the primary features (Wilson et al., 2019). Based on research from Kent et al. (2021), 18 speech dimensions were selected for DS. Darley et al. (1969) did not initially consider the features of unilateral upper motor neuron dysarthria. Additionally, the impact of apraxia of speech is considered for participants with a history of CVA.

## Conclusions

The SAP seeks to recruit, curate, and distribute a transcribed speech database that will accelerate research and development of ASR for people with speech disabilities. Preliminary results show that an ASR fine-tuned using 151 hr of speech from one set of people with PD (the SAP training set) can recognize the speech of other people with PD (the SAP unshared test set) with an error rate that is one third lower than that of a system trained using 960 hr of speech from people without PD.

## Data Availability Statement

The SAP 2023-10-05 Data Package, containing the curated speech of 211 participants with PD (the train and dev subsets described in this article), is currently available to universities and companies able to sign the data use agreement; see https://speechaccessibilityproject.beckman.illinois.edu/conduct-research-through-the-project.

The University of Illinois is responsible for collecting, hosting, and distributing the data set. Authors from other organizations contributed scientific expertise and oversight, but do not have direct oversight of the data set.

## Supplementary Material

10.1044/2024_JSLHR-24-00122SMS1 Supplemental Material S1 Examples of participant responses to the prompt ”Please explain the steps to making breakfast for 4 people.“

## Notes

**Publisher Note:** This article is part of the Forum: Research Symposium on Artificial Intelligence.
