# Supplementary material for: Community-Supported Shared Infrastructure in Support of Speech Accessibility
Source: J Speech Lang Hear Res. 2024 Sep 26;67(11):4162–75. doi: 10.1044/2024_JSLHR-24-00122 (PMC12379581; doi:10.1044/2024_JSLHR-24-00122)
Supplement: Supplemental Material S1 [file JSLHR-67-4162-s001.pdf]

Supplemental Material S1. Examples of participant responses to the prompt "Please explain the steps to making breakfast for 4 people."

Participant 4B8AAE89-754D-4C6E-F7DA-08DB65F35C8B:

"The breakfast I like to make for a group is Eggs Florentine. First, I make the hollandaise sauce the day before. And when it's time for breakfast, I poach the eggs and toast the english muffins. I put spinach on the english muffins, like (several uh let-) several pieces of spinach. Then I put the poached egg on top, reheat the hollandaise sauce, add a little lemon, and pour the hollandaise sauce over the eggs."

Participant C904EF6B-7796-4D27-DB5E-08DB263BD57D:

"The best way to make breakfast for four people is to ask them what they want, and then order it from a take out restaurant."
